# Opposing Effects of TGFβ and BMP in the Pulmonary Vasculature in Congenital Diaphragmatic Hernia

**DOI:** 10.3389/fmed.2021.642577

**Published:** 2021-03-11

**Authors:** Daphne S. Mous, Marjon J. Buscop-van Kempen, Rene M. H. Wijnen, Dick Tibboel, Rory E. Morty, Robbert J. Rottier

**Affiliations:** ^1^Department of Pediatric Surgery, Erasmus Medical Center – Sophia Children's Hospital, Rotterdam, Netherlands; ^2^Department of Cell Biology, Erasmus Medical Center, Rotterdam, Netherlands; ^3^Department of Lung Development and Remodelling, Max Planck Institute for Heart and Lung Research, Bad Nauheim, Germany; ^4^Department of Internal Medicine (Pulmonology), University of Giessen and Marburg Lung Center (UGMLC), Giessen, Germany

**Keywords:** lung, vasculature, BMP, TGF, congenital diagraphma hernia

## Abstract

**Background:** Pulmonary hypertension is the major cause of morbidity and mortality in congenital diaphragmatic hernia (CDH). Mutations in several genes that encode signaling molecules of the transforming growth factor β (TGFβ) and bone morphogenetic protein (BMP) pathways have previously been associated with CDH. Since studies on the activation of these pathways in CDH are scarce, and have yielded inconsistent conclusions, the downstream activity of both pathways was assessed in the nitrofen-CDH rat model.

**Methods and Results:** Pregnant Sprague-Dawley rats were treated with nitrofen at embryonic day (E) 9.5 to induce CDH in offspring. At E21, lungs were screened for the expression of key factors of both signaling pathways, at both the mRNA transcript and protein levels. Subsequently, paying particular attention to the pulmonary vasculature, increased phosphorylation of SMAD2, and decreased phosphorylation of Smad5 was noted in the muscular walls of small pulmonary vessels, by immunohistochemistry. This was accompanied by increased proliferation of constituent cells of the smooth muscle layer of these vessels.

**Conclusions:** Increased activation of the TGFβ pathway and decreased activation of the BMP pathway in the pulmonary vasculature of rats with experimentally-induced CDH, suggesting that the deregulated of these important signaling pathways may underlie the development of pulmonary hypertension in CDH.

## Introduction

Congenital diaphragmatic hernia (CDH) is a severe developmental anomaly characterized by a diaphragmatic defect. The concomitant pulmonary hypertension (PH) that develops in affected lungs can cause severe problems in the newborn, and is responsible for the high morbidity and mortality in these patients. Although the muscularization of the pulmonary vessels has been demonstrated to be increased in CDH (1), the pathophysiological basis of PH in these patients remains largely unclarified. Mutations in different genes involved in the transforming growth factor β (TGFβ) and bone morphogenetic protein (BMP) pathways have been described in both adult and pediatric patients with familial, heritable, and idiopathic pulmonary arterial hypertension (PAH). Of these genes, the BMP receptor 2 (BMPR2) is most commonly affected (2).

TGFβ is a negative regulator of airway branching in early lung development. However, TGFβ signaling is also active in the vascular and airway smooth muscle and alveolar and airway epithelium during late lung development. Both up- and down-regulation of TGFβ signaling impairs the alveolarization process (3, 4), depending on the period of study during gestation. Both TGFβ and BMP are documented to influence the proliferation of endothelial and smooth muscle cells, and control apoptosis and extracellular matrix secretion and deposition (5).

Studies on the TGFβ pathway in CDH have not yielded consistent conclusions. Decreased expression of TGFβ1 was found at the mRNA level in the hearts of the nitrofen-exposed rat pups with CDH (6), where increased expression of TGFβ1 in affected lungs was evident by immunohistochemistry (7). In contrast, other studies have reported no perturbations to TGFβ expression and activity—assessed by the phosphorylation of SMAD2/3—in both human samples as well as tissues harvested from the nitrofen-CDH rat model (8). A study performed in pregnant women carrying CDH fetuses revealed decreased TGFβ levels in the amniotic fluid, but no differences in expression of TGFβ in the lungs of these children after birth (9). The expression of both TGFβ receptor (TGFBR) 1 and 2 as well as endoglin, an auxiliary receptor of TGFβ, were found to be decreased in nitrofen-CDH rat pups (10).

**Figure 1:**
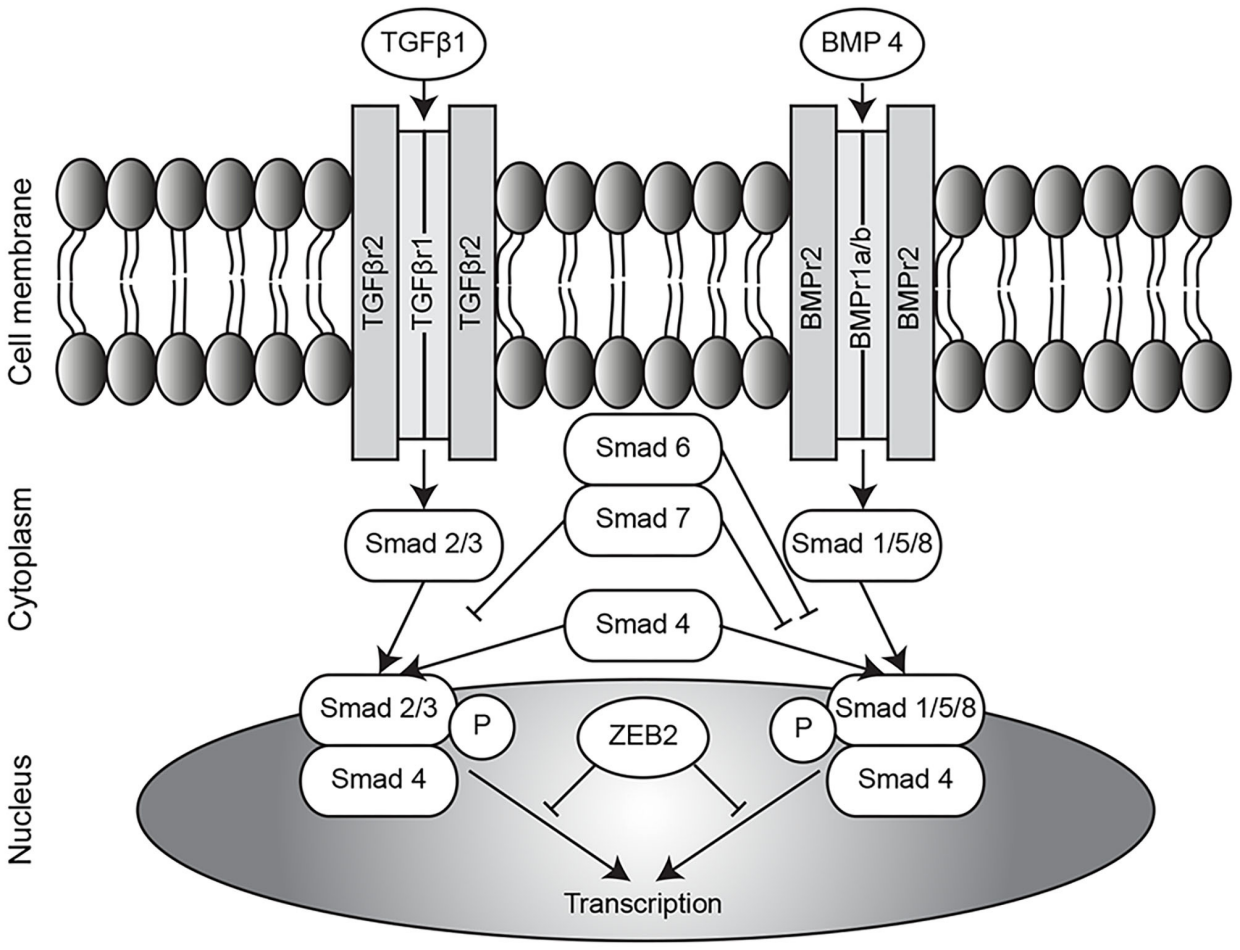
Overview of TGFβ/BMP pathway. Overview of the TGFβ and BMP pathways. TGFβ, transforming growth factor β; BMP, bone morphogenetic protein; ZEB2, zinc finger E-box binding homeobox 2; P, phosphorylation.

In contrast to the TGFβ pathway, conclusions drawn in several reports on components of the BMP pathway in CDH are consistent. Reduced expression of BMPR2 (11, 12) and BMP4 (12, 13) was found in the lungs of different animal models of CDH. Furthermore, the expression of apelin, a target gene of BMPR2 which can have a hypotensive function, is reported to be decreased in nitrofen-CDH rat pups (14); whilst expression of activin receptor-like kinase 1 (ALK1), another receptor of the BMP signaling pathway, was upregulated in the same animal model (15). However, Corbett et al. did not report any differences in downstream signaling of BMPR (16), and did not find any mutations in the BMPR2 gene in CDH patients (17). All findings reported to date addressing the TGFβ and BMP pathways in CDH is summarized in Supplementary Table 1 and an overview of both pathways is displayed in Figure 1.

Investigations conducted to date have focused largely on the expression of receptors in both the TGFβ and BMP pathways, but little is known about the actual activation of these pathways. Therefore, we hypothesized that the analysis of downstream mediators would identify changes in TGFβ and BMP signaling pathways in the lungs of rats in which CDH was induced by nitrofen exposure.

## Materials and Methods

### Animal Model

Pregnant Sprague-Dawley rats received either 100 mg nitrofen dissolved in 1 ml olive oil or just 1 ml olive oil by gavage on gestational age day E9.5. Nitrofen induces CDH in ~70% of the offspring, while all pups have pulmonary hypertension (18, 19). At embryonic day (E) 21, pups were delivered by cesarean section and euthanized by lethal injection of pentobarbital. Lung tissue of the CDH and control pups were isolated and processed for paraffin embedding (left lobes) or immediately snap frozen (right lobes) for protein and RNA analysis. All animal experiments were approved by an independent animal ethical committee and were conducted according to national guidelines.

### Quantitative Real-Time Polymerase Chain Reaction (qPCR)

RNA isolation, cDNA synthesis and subsequent qPCR analysis on right lung lobes was performed as previously (20). The gene-specific primers used are available upon request.

### Immunohistochemistry and Immunofluorescence Staining

Immunohistochemistry (IHC) was performed on 5-μm paraffin sections of the left lobe according to standard protocols, using the Envision™ detection system (Dako Cytomatic, Glostrup, Denmark) (20). Primary antibody used for IHC was ZEB2 [1:400, (21)]. Primary antibodies used for IF were smooth muscle actin (α-SMA; MS-113-P1; 1:500, Thermo Scientific, Fremont, CA, USA), phosphorylated SMAD 2 (pSMAD2; 1:250, Cell Signaling, Danvers, MA, USA), phosphorylated SMAD 1/5/8 (pSMAD1/5/8; 1:500, Kerafast, Boston, MA, USA), and KI-67 (1:100, Abcam, Cambridge, UK). Secondary antibodies against mouse (α-SMA) and rabbit (pSMAD2, pSMAD1/5/8, and KI-67) were used. Negative controls were performed by omitting the primary antibody. Antigen retrieval with citric acid buffer (pH 6.0) was used. Negative controls were performed by omitting the primary antibody.

### Immunoblotting

Snap-frozen right lung lobes were homogenized on ice in Carin buffer (20 mM Tris pH 8.0, 137 mM NaCl, 10 mM EDTA, 1% NP40, 10% glycerol), containing protease inhibitor Complete (Roche, Basel, Switzerland). Samples were centrifuged at 14,200 r.p.m. for 15 min and protein concentration in the supernatant was measured using the Bradford method. Subsequently 50 μg of protein per lane was loaded onto an SDS-PAGE and transferred to nitrocellulose membranes using wet blotting. Antigens were detected with TGFβ (1:1,000, Abcam), pSMAD2 (1:1,000, Cell Signaling), SMAD2 (1:1,000, Cell Signaling), pSMAD5 (1:1,000, Abcam), SMAD5 (1:1,000, Cell Signaling), and Zeb2 [1:1,000, (21)]. Cofilin (1:400, Abcam) and β-actin (1:1,000, Cell Signaling) were used for loading control.

### Statistical Analyses

Data are presented as percentages, means (SD) for normally distributed variables. Univariate analyses were performed using independent samples *t*-tests for normally distributed variables. The analyses were performed using SPSS 21.0 for Windows (Armonk, NY, USA: IBM Corp.). All statistical tests were two-sided and used a significance level of 0.05.

## Results

### TGFβ Activation Is Upregulated in CDH

**Figure 2:**
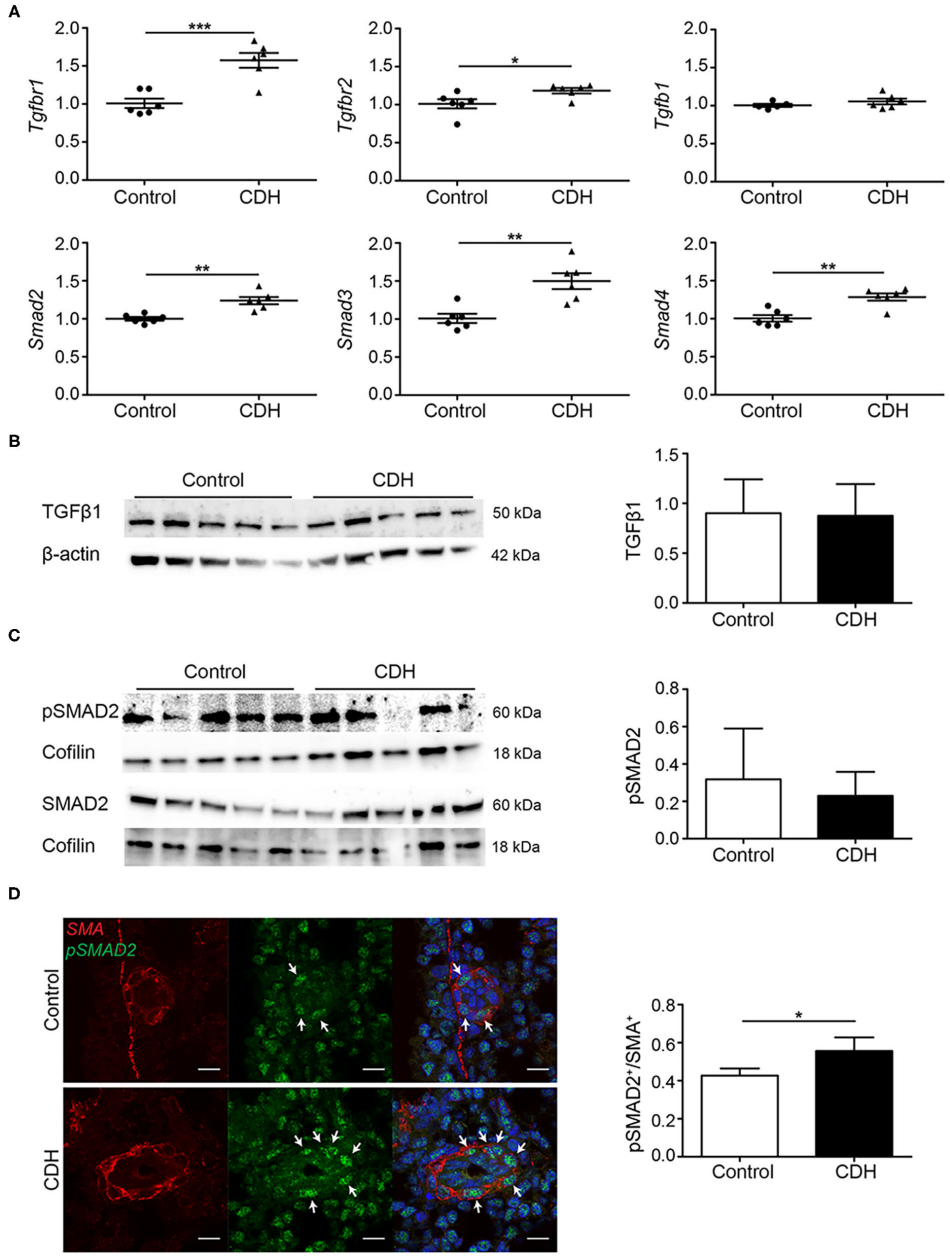
TGFβ activation is upregulated in experimental CDH. **(A)** Quantitative PCR revealed a significant increase in *Tgfbr1* and *Tgfbr2* in CDH (*p* < 0.001 and *p* = 0.033, respectively), but no difference in *Tgfb1* mRNA transcript abundance compared to control. The abundance of *Smad2, Smad3*, and *Smad4* mRNA transcripts were all significantly higher in CDH (*p* = 0.001, *p* = 0.002, and *p* = 0.002, respectively; *n* = 6 for both groups). **(B)** Western blot on whole lung homogenates revealed no differences in TGFβ1 abundance between control and CDH, when normalized to total protein amount using β-actin as a loading control (*n* = 5 for both groups). **(C)** The abundance of pSMAD2 was related to the total SMAD2 protein levels, which was not different between control and CDH in whole lung homogenates, where Cofilin was used as a loading control (*n* = 5 for both groups). **(D)** Representative images of immunofluorescence staining indicate an increase in the ratio of pSMAD2/SMA double-positive cells in small pulmonary vessels in CDH (*p* = 0.049; *n* = 3 samples for both groups). Six vessels per sample were counted. Scale bars represent 10 μm. *p < 0.05, **p < 0.01, ***p < 0.001. Error bars represent SD.

The expression of key signaling factors in the TGFβ pathway was assessed in whole lung homogenates at the mRNA level, where an increase in the abundance of mRNA transcript encoding both *Tgfbr1* and *Tgfbr2* receptors, but no difference in the abundance of the ligand *Tgfb1* mRNA transcript was noted. The abundance of mRNA transcripts encoding both the receptor-activated SMADs, *Smad2*, and *Smad3*, as well as the co-SMAD, *Smad4*, which together form a signaling complex for translocation into the cell nucleus, was increased in CDH (Figure 2A). No differences were found in expression of the TGFβ1 ligand at the protein level (Figure 2B). For the activation of the TGFβ pathway, receptor-activated SMADs must be phosphorylated. The degree of phosphorylation of SMAD2 was not different in whole lung homogenates of CDH pups compared to controls (Figure 2C). Since the abnormalities in the pulmonary vasculature are key pathological hallmarks of CDH, changes in SMAD phosphorylation were assessed in the small pulmonary vessels (25–50 μm) using immunofluorescence staining. This approach revealed an increased number of smooth muscle actin (SMA)-positive cells in the small vessels of CDH pups expressing phosphorylated SMAD2 (pSMAD2), which points to an increased activation of this pathway in the pulmonary vasculature (Figure 2D).

### BMP Activation Is Reduced in CDH

**Figure 3:**
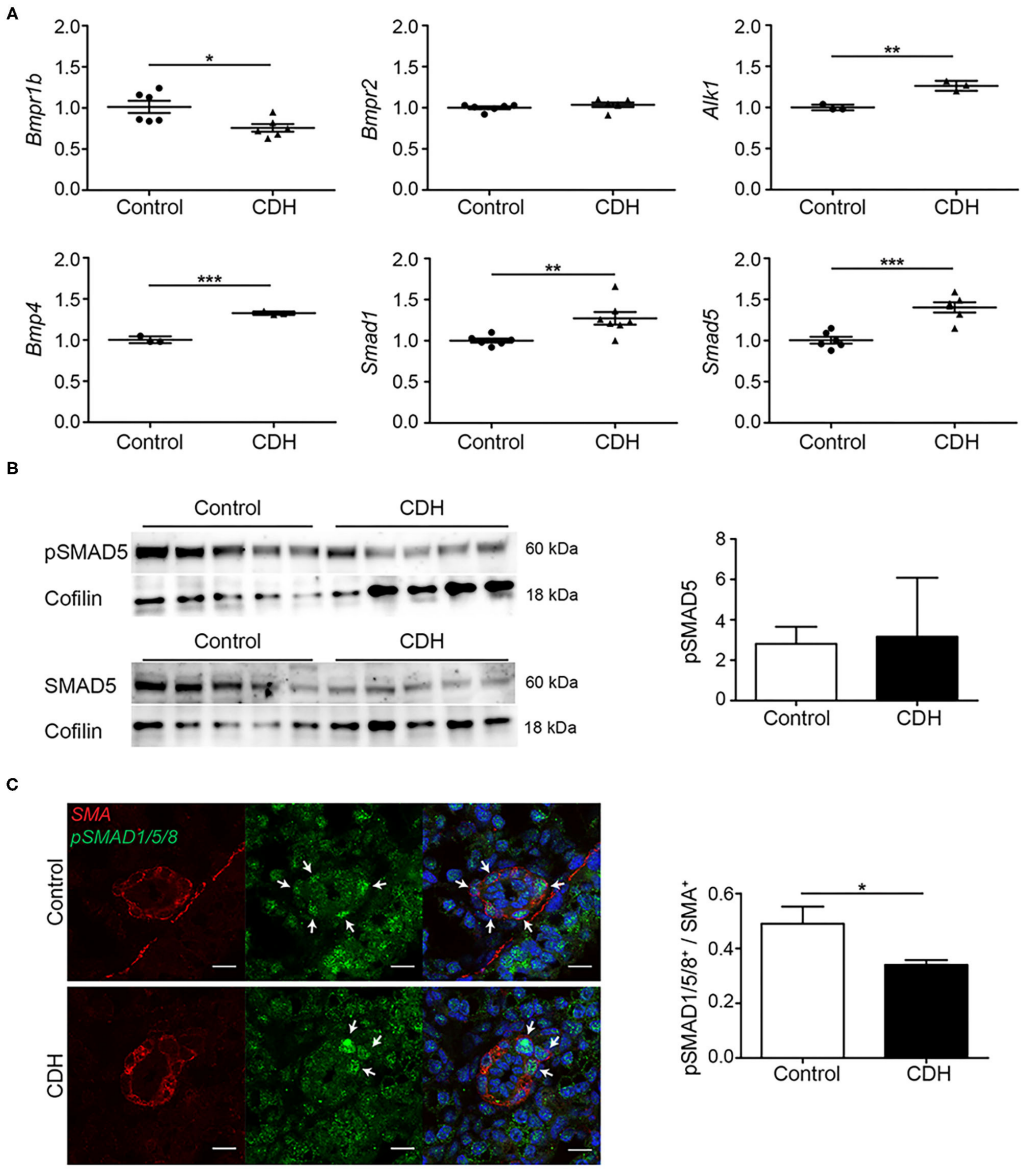
BMP activation is reduced in experimental CDH. **(A)** Quantitative PCR revealed a significantly decreased lung abundance of *Bmpr1b* (*p* = 0.016) but no differences in *Bmpr2*, and increased abundance of *Alk1* (*p* = 0.003) mRNA transcripts in CDH compared to control. The abundance of *Bmp4, Smad1*, and *Smad5* mRNA transcripts was significantly higher in CDH [*p* < 0.001, *p* = 0.009, and *p* < 0.001, respectively; *n* = 3 (*Alk1* and *Bmp4*) or 6 (rest) for both groups]. **(B)** The lung abundance of pSMAD5 was related to the total SMAD5 protein abundance, which was not different between control and CDH samples, where Cofilin was used as a loading control (*n* = 5 for both groups). **(C)** Representative images of immunofluorescence staining indicate a decrease in the ratio of pSMAD1/5/8/ SMA double-positive cells in small pulmonary vessels in CDH lungs (*p* = 0.016; *n* = 3 samples for both groups). Six vessels per sample were counted. Scale bars represent 10 μm. *p < 0.05, **p < 0.01, ***p < 0.001. Error bars represent SD.

In contrast to the TGFβ receptors, a decrease in *Bmpr1b* mRNA transcript abundance was noted in CDH, while no differences in the abundance of the well-studied *Bmpr2* were noted, comparing both groups at mRNA level in whole lung homogenates. Activin receptor-like kinase 1 (*Alk1*), another receptor in the BMP/TGFβ pathway which mediates the signal of *Bmp9* and *Bmp10*, was slightly increased in CDH. *Bmp4*, one of the important ligands in this pathway, and the receptor-activated *Smad1* and *Smad5* showed an increase in CDH (Figure 3A). Western blot on whole lung homogenates showed a decreased expression of SMAD5 in CDH with no differences in relative phosphorylation (Figure 3B). However, when focusing on the important pulmonary vasculature, the number of SMA positive cells expressing phosphorylated SMAD1/5/8 was reduced in CDH on immunofluorescence staining, indicating a decreased activation of this pathway in the pulmonary vasculature (Figure 3C).

### Downstream Effects of TGFβ and BMP Signaling

**Figure 4:**
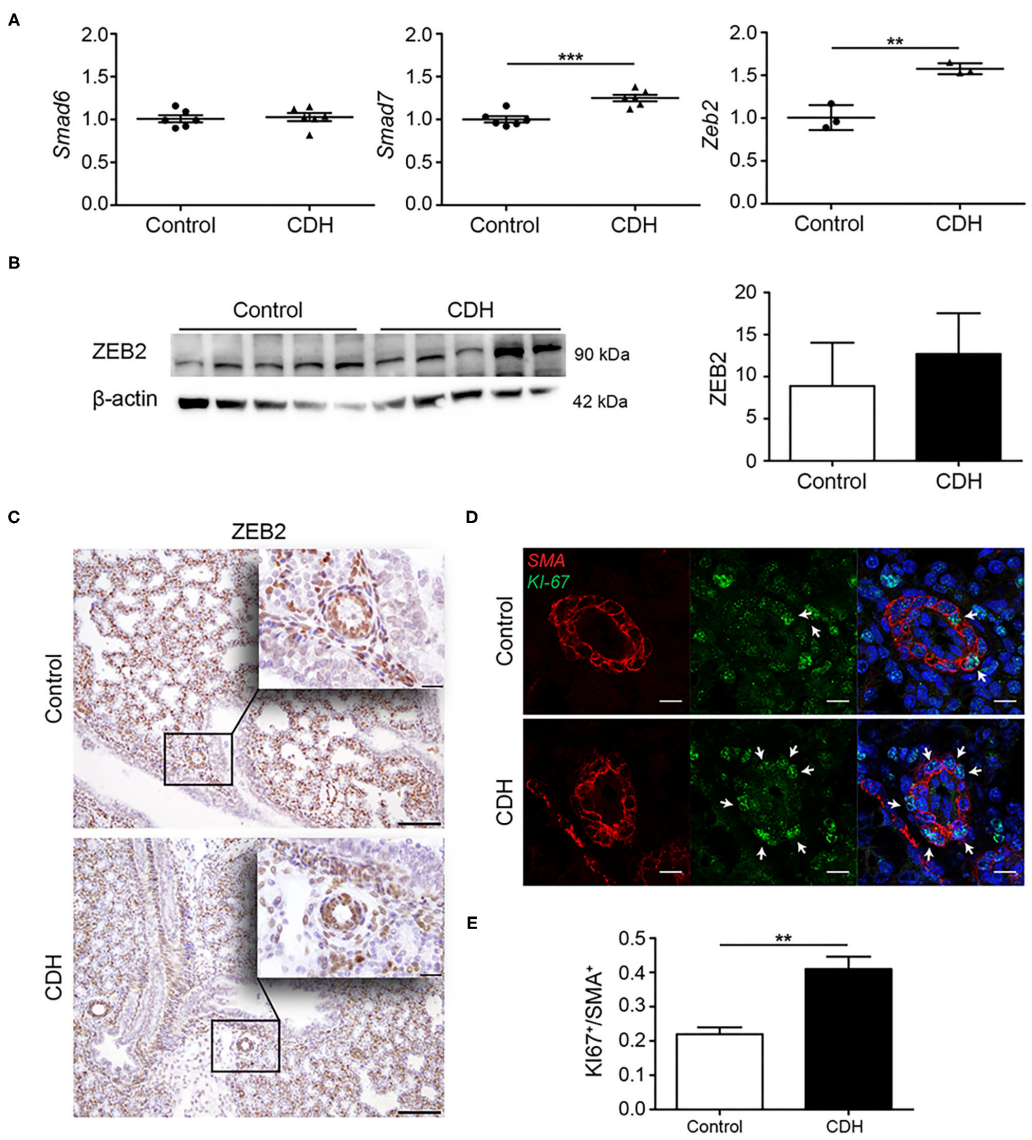
Modulators of TGFβ signaling are upregulated in CDH. **(A)** Quantitative PCR revealed no difference in the abundance of the inhibitory *Smad6* mRNA transcript, but increased abundance of inhibitory *Smad7* and *Zeb2* mRNA transcripts [*p* < 0.001 and *p* = 0.003, respectively; *n* = 3 (*Zeb2*) or 6 (*Smad6, Smad7*) per group; y-axis indicated fold-change]. **(B)** Western blot analyses of whole lung homogenates revealed no significant differences in ZEB2 protein abundance comparing CDH and control groups, where β-actin was used as a loading control, and as a reference for quantification (*n* = 5 for both groups). **(C)** Representative images of immunohistochemistry staining show no differences in expression of ZEB2 in the small vessels of all lungs (*n* = 3 samples for both groups). Scale bars represent 100 μm (low power) and 20 μm (high power). **p < 0.01, ***p < 0.001. Error bars represent SD. **(D)** Increased proliferation of the muscular vessel wall in CDH. Representative images of immunofluorescence staining revealed an increase in KI-67/SMA double-positive cells in small pulmonary vessels in CDH (*p* = 0.001; *n* = 3 samples for both groups). **(E)** Quantification of proliferative SMA+ cells. Four vessels per sample were counted. Scale bars represent 10 μm. **p < 0.01, ***p < 0.001. Error bars represent SD.

Both the TGFβ and BMP pathways can be inhibited by the inhibitory SMADs, SMAD6, and SMAD7. These proteins compete with SMAD4 in the formation of heteromeric signaling complexes and can, therefore, prevent transcription of target genes. No differences were noted in the expression of *Smad6* at the mRNA level, but lung *Smad7* transcript abundance was increased in CDH. The lung abundance of mRNA transcripts encoding *Zeb2*, a transcriptional corepressor of the activated pathway, were increases in CDH at the mRNA level (Figure 4A). However, no significant differences in protein levels of ZEB2 were noted by western blot analysis of whole lung homogenates (Figure 4B), and no changes in the expression of ZEB2 were noted in the small vessels using immunohistochemistry (Figure 4C). Since increased activation of the TGFβ pathway can induce proliferation of pulmonary artery smooth muscle cells, the expression of KI-67, a marker for proliferation, was used to identify proliferating cells in the vascular wall. In small pulmonary vessels in CDH, more SMA-positive cells expressed KI-67 (Figures 4D,E).

## Discussion

In this report, upregulated activation of the TGFβ pathway and downregulated activation of the BMP pathway in small pulmonary vessels in the nitrofen-CDH rat model are demonstrated at the cellular level.

No differences were observed in the ligand TGFβ1 and the degree of phosphorylation of both SMAD2 and SMAD5 at the protein level in whole lung homogenates. Although the total amount of SMAD5 and pSMAD5 was less in whole lung homogenates of CDH pups, no changes were noted in the degree of phosphorylation in total lung extracts. At the cellular level, however, the smooth muscle layer of the small pulmonary vessels of nitrofen-CDH pups revealed increased abundance of pSMAD2 and decreased abundance of pSMAD1/5/8, indicative of more active TGFβ signaling and reduced BMP signaling, respectively. The latter is in line with Makenga and colleagues who reported decreased pSMAD1/5/8 in CDH lung homogenates. Moreover, the differences observed in the small pulmonary arteries in CDH lungs may also reflect the fact that the perivascular cells in CDH lungs are more differentiated compared to perivascular cells in control lungs (22).

Phosphorylation of the receptor-activated SMADs is necessary for the activation of downstream mediators and, therefore, plays an important role in pathway activation. The increased expression of the inhibitory *Smad7* and corepressor *Zeb2* at the mRNA level is in line with SMAD7 being a direct target of ZEB2 and may point to increased production of these inhibitors in order to inhibit the increased activity of the TGFβ pathway (23). The absence of any observed changes in *Smad6*, which only inhibits the BMP pathway, strengthens this idea. However, the expression of ZEB2 at the protein level in whole lung homogenates exhibited a trend toward an increase, and no differences were noted by immunostaining of the pulmonary vessels, indicating a discrepancy between RNA and protein expression. The latter could, in part, explain differences between several reports on TGFβ and BMP signaling in CDH. Moreover, the usage of specific parts of the lung or isolated lung cells may also lead to differences or even opposing results between different reports. Both TGFβ and BMP can regulate proliferation of vascular cells and previous studies have reported increased proliferation of pulmonary artery smooth muscle cells from patients with PAH in response to TGFβ1 (24, 25). The increased proliferation of constituent cells of the smooth muscle layer of small pulmonary vessels was noted in the present study in nitrofen-CDH pup lungs, which might indicate an abnormal response of these cells to the increased TGFβ activity.

TGFβ is a target of retinoic acid (RA) (26), and increased activity of the TGFβ pathway with higher levels of pSMAD2 has been described in RA-deficient foreguts, and in a mouse model with RA deficiency. In that study, lung agenesis was observed both by decreasing RA levels as well as by increasing TGFβ levels, indicating the interaction between both pathways early in development (27). Furthermore, a study in rats with alveolar hypoplasia caused by caloric restriction exhibited improvement of alveolar formation after treatment with RA, accompanied by a decrease in TGFβ activity at postnatal day 21 (28). These findings strengthen the results presented here, about increased TGFβ activity in nitrofen-exposed rats, where nitrofen has been reported to disrupt the retinoid signaling pathway (29). Since a reduction in retinol and retinol binding protein (RBP) has been found in human newborns with CDH (30, 31), and several key components of the RA pathway are affected in human and experimental CDH (32), the increased activity of the TGFβ pathway might play an important role in the development of the lungs in CDH.

Studies available in the literature have reported conflicting trends in expression of different signaling factors in CDH, which might be explained by the differences in the gestation of the animals under study. In the present study, some variability between samples was also noted, suggesting that small differences in gestational age may have an appreciable impact on trends in the expression of signaling molecules under study.

We initially analyzed the TGF-β and BMP pathways in whole lungs, and given our previous report on vascular abnormalities in CDH (22), we focused on the activation of the TGF-β and BMP pathways in the vasculature, using immunofluorescence staining, showing a clearly difference in phosphorylation of SMAD2 and SMAD5.

In conclusion, increased phosphorylation of SMAD2 and decreased phosphorylation of SMAD5 was noted in the in the vessel walls of small pulmonary vessels of nitrofen-CDH pups. These data indicate increased activation of the TGFβ pathway and decreased activation of the BMP pathway in the pulmonary vasculature of these animals at day 21 of gestation, possibly leading to increased proliferation of the muscularized vessel wall. Since the different factors in these pathways are differently expressed during gestation and might differ from the human situation, further research must be conducted at different developmental stages, and most importantly, in material of human patients.

## Data Availability Statement

The original contributions presented in the study are included in the article/Supplementary Material, further inquiries can be directed to the corresponding authors.

## Ethics Statement

The animal study was reviewed and approved by independent animal ethical committee (EMC).

## Author Contributions

DT, RM, and RR: conceptualization and writing—original draft. DM, MB-vK, RM, and RR: data curation. DM, MB-vK, and RR: formal analysis and methodology. RM and RR: funding acquisition and supervision. DM and MB-vK: investigation and validation. DT and RW: resources. DM, MB-vK, RW, DT, RM, and RR: writing—review and editing. All authors contributed to the article and approved the submitted version.

## Conflict of Interest

The authors declare that the research was conducted in the absence of any commercial or financial relationships that could be construed as a potential conflict of interest.
