# Supplementary material for: Opposing Effects of TGFβ and BMP in the Pulmonary Vasculature in Congenital Diaphragmatic Hernia
Source: Front Med (Lausanne). 2021 Mar 11;8:642577. doi: 10.3389/fmed.2021.642577 (PMC7991367; doi:10.3389/fmed.2021.642577)
Supplement: Supplementary file 1 [file Table_1.DOCX]

**Supplementary table 1**

| **Factor** | **Animal** | **Human** |
| --- | --- | --- |
| TGFβ | Decreased ([6](#_ENREF_6)) | Decreased ([9](#_ENREF_9)) |
|  | Increased ([7](#_ENREF_7)) | No difference ([8](#_ENREF_8)) |
|  | No difference ([8](#_ENREF_8)) |  |
| TGFβ receptor | Decreased ([10](#_ENREF_10)) |  |
| BMPr2 | Decreased ([12](#_ENREF_12)) | No difference ([17](#_ENREF_17)) |
|  | Decreased ([11](#_ENREF_11)) |  |
| BMP4 | Decreased ([13](#_ENREF_13)) |  |
|  | Decreased ([12](#_ENREF_12)) |  |
| ALK1 | Increased ([15](#_ENREF_15)) |  |
| pSm 1/5/8 | No difference ([16](#_ENREF_16)) |  |
| Apelin | Decreased ([14](#_ENREF_14)) |  |
